# Red blood cell transfusion and increased length of storage are not associated with deep vein thrombosis in medical and surgical critically ill patients: a prospective observational cohort study

**DOI:** 10.1186/cc10526

**Published:** 2011-11-02

**Authors:** Christina Katsios, Lauren Griffith, Philip Spinella, Jacques Lacroix, Mark Crowther, Paul Hebert, Maureen Meade, William Geerts, Christian Rabbat, Deborah Cook

**Affiliations:** 1 Department of Medicine, McMaster University, 1200 Main Street West, Hamilton, ON L8S 4L8, Canada; 2 Blood Systems Research Institute, 270 Masonic Avenue, San Francisco, CA 94118, USA; 3 Hopital St Justine, 3175, Chemin de la Côte-Sainte-Catherine, Montreal, QC H3T 1C5, Canada; 4 Ottawa Hospital Research Institute, University of Ottawa, 725 Parkdale Avenue, Ottawa, ON K1Y 4E9, Canada; 5 Department of Medicine, University of Toronto, Suite RFE 3-805, 200 Elizabeth Street, Toronto, ON M5G 2C4, Canada

## Abstract

**Introduction:**

With prolonged storage times, cell membranes of red blood cells (RBCs) undergo morphologic and biochemical changes, termed 'RBC storage lesions'. Storage lesions may promote inflammation and thrombophilia when transfused. In trauma patients, RBC transfusion was an independent risk factor for deep vein thrombosis (DVT), specifically when RBC units were stored > 21 days or when 5 or more units were transfused. The objective of this study was to determine if RBC transfusions or RBC storage age predicts incident DVT in medical or surgical intensive care unit (ICU) patients.

**Methods:**

Using a database which prospectively enrolled 261 patients over the course of 1 year with an ICU stay of at least 3 days, we analyzed DVT and RBC transfusions using Cox proportional hazards regression. Transfusions were analyzed with 4 thresholds, and storage age using 3 thresholds. DVTs were identified by twice-weekly proximal leg ultrasounds. Multivariable analyses were adjusted for 4 significant DVT predictors in this population (venous thrombosis history, chronic dialysis, platelet transfusion and inotropes).

**Results:**

Of 261 patients, 126 (48.3%) had at least 1 RBC transfusion; 46.8% of those transfused had ≥ 5 units in ICU. Patients receiving RBCs were older (68.8 vs 64.1 years), more likely to be female (47.0 vs 30.7), sicker (APACHEII 26.8 vs 24.4), and more likely to be surgical (21.4 vs 8.9) (*P *< 0.05). The total number of RBCs per patient was 1-64, mean was 6.3 (SD 7.5), median was 4 (IQR 2,8). In univariate analyses, there was no association between DVT and RBC exposure (1 day earlier, 3 days earlier, 7 days earlier, or ever) or RBC storage (≤ 7 or > 7 days, ≤ 14 or > 14 days, ≤ 21 or > 21 days). Among patients transfused, no multivariable analyses showed that RBC transfusion or storage age predicted DVT. Trends were counter to the hypothesis (e.g., RBC storage for ≤ 7 days suggested a higher DVT risk compared to > 7 days (HR 5.3; 95% CI 1.3-22.1).

**Conclusions:**

We were unable to detect any association between RBC transfusions or prolonged red cell storage and increased risk of DVT in medical or surgical ICU patients. Alternate explanations include a lack of sufficient events or patients' interaction, between patient groups, a mixing of red cell storage times creating differential effects on DVT risk, and unmeasured confounders.

## Introduction

Over 24 million units of packed red blood cells (RBCs) were transfused in the US in 2004. More than 40% of critical care patients receive RBC transfusions while in the intensive care unit (ICU) [1,2]. Numerous studies demonstrate that RBC transfusions are associated with complications in critically ill patients, including volume overload, acute lung injury, hemolytic reactions, increased risk of infection, multiple organ dysfunction, and death [3-6].

Units of packed RBCs can be stored for up to 42 days after donation. With increasing age, the membranes of RBCs undergo morphologic and biochemical changes, termed 'RBC storage lesions' [7]. Storage produces a predictable change in morphology - starting from the deformable biconcave disc to a reversibly deformed ecchinocyte to an irreversibly deformed spheroecchinocyte [8]. In addition to these morphologic changes and their consequences, predictable biochemical and oxidative changes occur over prolonged storage. Cardo and colleagues [9] showed that stored RBCs have membrane biochemical changes, such as upregulated phosphatidylserine on their surface, that may promote inflammation and a tendency toward thrombosis [8,9]. RBC membrane microparticle production increases with storage duration, and these bioactive lipids have been demonstrated to have proinflammatory, immune-suppressive, and procoagulant properties [10]. A prior study has also demonstrated that immumodulation [6] and impaired vasoregulation occurred with prolonged storing of units and that poor tissue perfusion was a result [11].

RBC transfusion was found to be an independent risk factor for deep vein thrombosis (DVT) in 716 trauma patients (odds ratio 1.74, 95% confidence interval (CI) 1.03 to 2.93) [12,13]. Duration of storage may modify this risk. In a study of 202 trauma patients transfused more than 5 units of RBCs, DVT rates were lower among patients receiving RBCs stored fewer than 28 days compared with more than 28 days (16.7% versus 34.5%, *P *= 0.006); mortality was also higher among patients receiving blood with longer storage times (13.9% versus 26.7%, *P *= 0.02) [14]. Conflicting evidence exists, and, to date, no transfusion policy changes in regard to the age of blood have been made or are warranted on the basis of the data currently available. Recent feasibility studies cite a potential negative impact on RBC availability if RBC age transfusion policies are implemented [15]. Furthermore, there is no current evidence of the relationship between age of blood and units of transfusion in medical or surgical ICU patients. These patients represent a new demographic and some of them will have lower APACHE II (Acute Physiology and Chronic Health Evaluation II) scores than the trauma population. If storage lesions are indeed thrombophilic, the age of blood may present an increased risk of DVT irrespectively of the patient population. The objective of this study was to determine whether either the number of RBC transfusions or the age of RBCs transfused was a predictor of the development of DVT in medical or surgical ICU patients.

## Materials and methods

### Study population

We used a prospective study database of 261 critically ill patients admitted to an academic, 15-bed ICU from January 2001 to January 2002. Inclusion criteria were age of more than 18 years and an expected ICU stay of greater than 72 hours. Patients were excluded if they had an admitting diagnosis of trauma or orthopedic surgery or if they were pregnant or expected to undergo withdrawal of life support. Patients were followed until death or discharge from the hospital. Substitute decision makers or patients provided written informed consent to participate. The research ethics board of St. Joseph's Hospital (Hamilton, ON, Canada) approved this protocol.

In the original study database, we had collected baseline characteristics such as age, sex, admission diagnosis, APACHE II score, and organ function. During the ICU stay, data was collected on transfusion history, advanced life support measures and VTE risk factors. The restrictive RBC transfusion threshold from the TRICC (Transfusion Requirements in Critical Care) trial [3] was in place as the standard of care in this hospital at the time of this study. That is, the transfusion trigger was a hemoglobin target of 70 g/dL unless patients were actively bleeding or had coronary ischemia. For purposes of this study on the relation between RBC transfusion and DVT, one of the investigators (CK) retrospectively abstracted additional data on these patients from the medical record, Transfusion Medicine databases (LifeLine; Mediware Information Systems Inc., Lenexa, KS, USA), and Canadian Blood Services logs. The duration of storage for each RBC unit and the total number of units transfused were documented. Reliability was ensured by the consistency of the two databases. Transfused units were not leukodepleted at this time. At the time of this study, Canadian Blood Services did not have policies about the age of blood. However, the standard of practice in our blood bank is to issue the oldest available unit of electronically crossmatch-confirmed blood.

### Deep vein thrombosis diagnosis

Twice-weekly lower limb compression ultrasounds were used to diagnose proximal DVT and were performed in all patients. A DVT was defined as a non-compressible venous segment detected by ultrasound in any of the following six sites: trifurcation of the deep calf veins, distal popliteal vein, proximal popliteal vein, distal femoral vein, midfemoral vein, and common femoral vein. The ultrasound report noted whether the vessel was fully compressible, fully compressible with intraluminal filling defects, not fully compressible, or not visualized. Patients with positive or indeterminate ultrasound underwent bilateral lower extremity ascending contrast venography unless they had absolute contraindications. Investigations for thrombosis at other anatomical sites were performed only if clinically indicated.

To ensure uniformity of DVT screen ultrasounds, two trained ultrasonographers performed all tests independently of each other and were blinded to patient history and physical examinations. A previous inter-rater reliability study demonstrated perfect agreement between these two ultrasonographers at all six venous sites [16].

To avoid overestimation of DVT incidence because of an omission of DVT prophylaxis, a DVT prophylaxis regimen was instituted. Patients received 5,000 units of unfractionated heparin subcutaneously twice daily. If there was a contraindication to heparin, pneumatic compression stockings or antiembolic stockings were used.

### Statistical analysis

We examined the association of DVT with RBC transfusions by using Cox proportional hazards regression and time-dependent covariates. Time-dependent covariates allow for a change in risk profiles over time. For example, a patient, in theory, could be at higher risk if a transfusion was given on the previous day but not if the transfusion was given a day or more before that. The temporal relationship was analyzed with four thresholds (transfusion 1 day earlier, 3 days earlier, 7 days earlier, or ever before). Transfused RBC storage age was analyzed by using three sliding thresholds (not more than 7 or more than 7 days, not more than 14 or more than 14 days, and not more than 21 or more than 21 days). Those patients who did not receive a transfusion were included with the 'lower risk' shorter storage group in regression analyses. If a patient received more than one RBC unit on a single day, we categorized the patient on the basis of their oldest unit.

Independent variables included were baseline factors (patient age, sex, and body mass index); admission diagnosis; medical or surgical admitting diagnosis; APACHE II score; pre-ICU location; personal or family history of venous thromboembolism (VTE) disease; known thrombophilic disorder; active malignancy (defined as active malignancy or treatment for active malignancy within the previous 6 months); chronic cardiac, respiratory, renal, or central nervous system disease; smoking; hospitalization within the previous 6 months; surgery within the previous 12 weeks; bed rest for at least 3 days in the previous 4 weeks; and activity level (totally independent, somewhat limited, or severely limited). Additional factors included exposures documented within 2 weeks before ICU admission and daily thereafter (central venous catheters, subcutaneous unfractionated heparin, heparin for catheter patency, therapeutic unfractionated heparin or low-molecular-weight heparin, warfarin, acetylsalicyclic acid, non-steroidal anti-inflammatory drugs, other anti-platelet drugs, vitamin K, RBCs, fresh frozen plasma, platelets, and cryoprecipitate). No patient received recombinant factor VIIa during this study.

Additional independent variables included daily ICU parameters such as the multiorgan dysfunction score, mechanical ventilation, vasopressors/inotropes, dialysis, surgical interventions, hemoglobin, platelet count, international normalized ratio, partial thromboplastin time, and intravenous bolus or continuous infusion of sedatives, paralytics, or opiates.

We first conducted a univariable analysis testing the association between RBC transfusion and RBC storage time with DVT. We then conducted multivariable Cox proportion analyses, which were adjusted for the four previously identified significant DVT risk factors in this population (history of previous VTE, end-stage renal disease requiring chronic dialysis, platelet transfusion, and inotropes/vasopressors) [17,18]. For the final model, we calculated hazard ratios and 95% CIs for all variables significant at a *P *value of less than 0.05. We tested for interactions among significant factors in the final model. We also evaluated whether risk factors varied over the ICU stay by using a non-proportional hazard Cox model and testing the interaction of each variable with time. We used the computer program SAS (statistical analysis software) (SAS Institute Inc., Cary, NC, USA).

## Results

**Figure 1:**
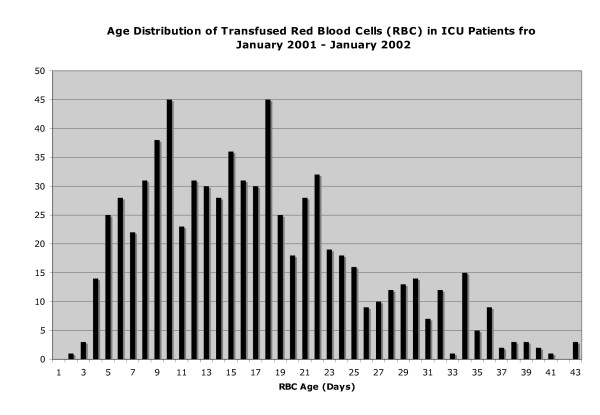
**Age distribution of transfused packed red blood cell (RBC) units in storage**. The age distribution for all units of RBCs transfused to patients in this study is shown. ICU, intensive care unit.

**Figure 2:**
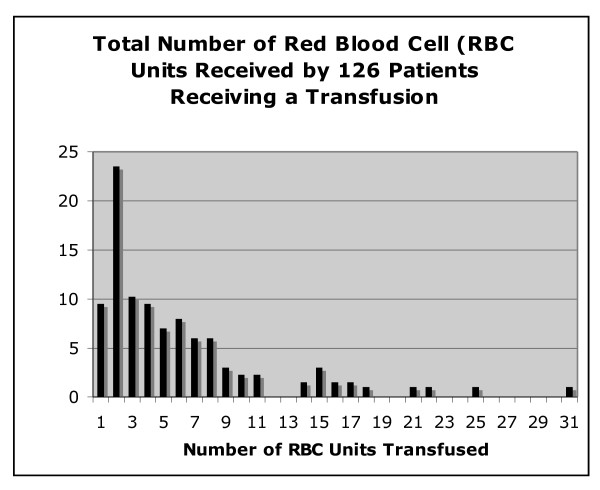
**Total number of transfused packed red blood cell (RBC) units**. The total number of RBC units transfused in patients during their intensive care unit (ICU) stay is shown. Data are truncated at 31 units. One patient receiving 64 units is not depicted in the figure.

Of 261 patients, 126 (48.3%) had at least one RBC transfusion during their ICU stay (Figure 1). Over the course of their ICU stay, 46.8% of the patients who were transfused received 5 or more units (Figure 2). Patients who were transfused were older (68.8 versus 64.1 years), more likely to be female (47.0% versus 30.7%), sicker (APACHE II score of 26.8 versus 24.4), and more likely to have a surgical event precipitating their ICU stay (21.4% versus 8.9%) (all *P *< 0.05). The total number of RBCs transfused per patient ranged from 1 to 64, the mean was 6.3 (standard deviation of 7.5), and the median was 4 (interquartile range of 2 to 8). Per transfusion episode, the mean number of units transfused was 2.4. Of the 261 patients, 241 were included in our analysis as we were interested in time-dependent covariates; so patients with only 1 study day in the ICU or with a prevalent DVT or pulmonary embolus were excluded from the analysis. Baseline demographics are presented in Table 1.

**Table 1:** Baseline characteristics of patients in regression analysis

Characteristic	All (*n *= 241)	RBCs (*n *= 117)	No RBCs (*n *= 124)	*P *value
Age in years, mean (SD)	66.4 (15.2)	68.8 (13.3)	64.1 (16.5)	0.02
Females, number (percentage)	93 (38.6)	55 (47.0)	38 (30.7)	0.009
APACHE II score, mean (SD)	25.5 (8.4)	26.8 (8.3)	24.2 (8.3)	0.02
Multiple organ dysfunction score, mean (SD)	6.3 (3.9)	7.6 (3.8)	5.1 (3.6)	< 0.001
Height in centimeters, mean (SD)	168.7 (10.7)	166.7 (11.4)	170.5 (9.7)	0.007
Weight in kilograms, mean (SD)	76.5 (19.6)	75.4 (19.3)	77.7 (19.9)	0.37
Body mass index in kg/m^2^, mean (SD)	27.1 (7.2)	27.4 (7.9)	26.8 (6.4)	0.53
Medical admission, number (percentage)	183 (75.9)	75 (64.1)	108 (87.1)	< 0.001
Primary admission diagnosis category, number (percentage)				
Pulmonary	89 (36.9)	31 (26.5)	58 (46.8)	< 0.001
Cardiovascular	62 (25.7)	37 (31.6)	25 (20.2)	
Gastrointestinal	37 (15.4)	24 (20.5)	13 (10.5)	
Renal	17 (7.1)	12 (10.3)	5 (4.0)	
Neurological	16 (6.6)	2 (1.7)	14 (11.3)	
Sepsis	12 (5.0)	7 (6.0)	5 (4.0)	
Other^a^	8 (3.3)	4 (3.4)	4 (3.2)	
Location prior to admission, number (percentage)				
Ward	106 (44.0)	53 (45.3)	53 (42.7)	0.002
Emergency room	60 (24.9)	18 (15,4)	42 (33.9)	
Operating room	36 (14.9)	25 (21.4)	11 (8.9)	
Other hospital ICU	39 (16.2)	21 (18.0)	18 (14.5)	
Central venous catheter, number (percentage)	206 (85.5)	110 (94.0)	96 (77.4)	< 0.001
Mechanical ventilation, number (percentage)	218 (90.5)	110 (94.0)	108 (87.1)	0.07
Days of mechanical ventilation, median (IQR)	7 (3, 15)	12 (6, 23)	4 (2, 9)	< 0.001
Days in ICU, median (IQR)	10 (6, 20)	16 (8, 26)	8 (5, 13)	< 0.001
ICU mortality, number (percentage)	66 (27.4)	40 (34.2)	26 (21.0)	0.02
Days in hospital, median (IQR)	25 (13, 54)	30 (15, 64.5)	19 (11, 41)	0.002
Hospital mortality, number (percentage)	94 (39.0)	55 (47.0)	39 (31.5)	0.01

The clinical characteristics of 241 medical-surgical critically ill patients who underwent twice-weekly lower extremity ultrasounds to screen for proximal deep vein thrombosis and who were included in the regression analysis are shown. ^a^'Other' refers to necrotizing fasciitis (3), diabetic ketoacidosis (1), facial surgery (1), Addison disease (1), mushroom poisoning (1), and post-partum hemorrhage (1). APACHE II, Acute Physiology and Chronic Health Evaluation II; ICU, intensive care unit; IQR, interquartile range; RBC, packed red blood cell unit; SD, standard deviation.

Occlusive proximal leg DVT was identified in 7 patients, and 7 patients had evidence of chronic venous abnormalities (consistent with prior DVT) on ICU admission. During their ICU stay, an additional 25 patients developed proximal leg DVT, ultimately yielding an incidence of 9.6%. Of the 7 acute prevalent and 25 acute incident DVTs, 3 were catheter-related secondary to femoral venous access.

We first conducted a univariable analysis (Table 2) testing the association between RBC transfusion and RBC storage time with DVT, which was non-significant. In univariable analyses, there was no association between DVT and RBC temporal exposure to RBCs (1 day earlier, 3 days earlier, 7 days earlier, or ever) or RBC storage age (not more than 7 or more than 7 days, not more than 14 or more than 14 days, or not more than 21 or more than 21 days). Among patients transfused, no multivariable analyses (Table 3) showed that RBC transfusion or age predicted DVT. Findings were counter to the hypothesis generated by prior clinical studies; for example, RBC storage for not more than 7 days suggested a higher DVT risk compared with more than 7 days (hazard ratio 5.3, 95% CI 1.3 to 22.1).

**Table 2:** Packed red blood cell unit storage age and deep vein thrombosis incidence: univariable analyses

	Temporal relationship to DVT			
Age of RBC units	Previous day	Previous 3 days	Previous week	Ever before
Any age	1.7 (0.6, 4.4)	1.0 (0.4, 2.4)	1.4 (0.6, 3.3)	1.4 (0.6, 3.3)
≤ 7 days	4.5 (0.6, 35.1)	2.0 (0.3, 15.5)	4.6 (1.3, 17.0)	3.6 (0.9, 14.3)
> 7 days	1.3 (0.4, 4.1)	0.8 (0.3, 2.2)	1.0 (0.4, 2.5)	1.2 (0.5,3.1)
≤ 14 days	1.9 (0.4, 8.4)	1.0 (0.2, 4.2)	1.4 (0.5, 4.3)	1.9 (0.7, 5.1)
> 14 days	1.4 (0.4, 5.0)	0.8 (0.3, 2.5)	1.2 (0.5, 3.0)	1.2 (0.5, 3.1)
≤ 21 days	1.8 (0.6, 5.7)	1.2 (0.4, 3.3)	1.7 (0.7, 4.3)	2.0 (0.8, 5.1)
> 21 days	1.0 (0.1, 7.6)	0.4 (0.1, 3.1)	1.4 (0.5, 4.3)	1.3 (0.4, 3.9)

Data of the 241 patients analyzed and the temporal packed red blood cell (RBC) transfusion thresholds are shown. We present the hazard ratios (with 95% confidence intervals) for the association between risk of developing a deep vein thrombosis (DVT) in the intensive care unit (ICU) and RBC storage times. This association was determined by using four sets of data: (1) the data on the previous ICU day to predict DVT, (2) data from any time in the previous 3 days, (3) data from any time in the previous week in the ICU to predict DVT, and (4) data from any prior ICU day to predict DVT.

**Table 3:** Red blood cell unit storage age and incidence of deep vein thrombosis: multivariable analysis

Age of transfused blood	Hazard ratio (95% CI)	*P *value
≤ 7 days	5.3 (1.3, 22.1)	0.12
> 7 days	1.0 (0.4, 2.9)	
≤ 14 days	1.8 (0.6, 5.1)	0.59
> 14 days	1.2 (0.4, 3.2)	
≤ 21 days	2.1 (0.8, 5.5)	0.33
> 21 days	0.9 (0.3, 2.8)	

The hazard ratios with 95% confidence intervals (CIs) for the association between age of blood and acquired risk of deep vein thrombosis in the intensive care unit are shown.

## Discussion

In our study, we found that neither the number of transfused RBC units nor the duration of RBC storage affected DVT risk in medical or surgical ICU patients. Although inflammatory membrane lipid changes on stored RBCs facilitate thrombin generation, leading stored RBCs to potentially contribute to a hypercoagulable state [9], RBCs stored for not more than 7 days in our study were associated with a trend toward a higher risk of DVT compared with RBCs stored more than 7 days.

Possible explanations for our findings include insufficient power - owing to the relatively low DVT rate - to detect an association if one truly existed. However, clinically important values are included within our CIs. For example, the prevalence and incidence of DVT in our study were 3% and 10%, respectively. A previous study found that 32% to 40% of ICU patients developed a DVT [19]. Other potential explanations for the lack of association between RBC transfusion and DVT in this study include unmeasured confounders, a difference between effects on DVT risk in medical or surgical patients and those in trauma patients, differences in definition of prolonged storage, and a lack of a true relationship. Also, we did not include RBC transfusions in the hospital stay prior to ICU admission. Because our study design is different from that of the two trauma studies [12,14], direct comparisons are difficult to make. Another limitation of our study may be accuracy of the retrospective collection of the storage age of the transfused RBC units; storage age was obtained by one data collector and was not verified in duplicate.

The average diameter of RBCs is 7.2 μm, whereas capillary diameter is 3 to 8 μm. Normal RBCs successfully pass through capillary beds because of the ability to change shape; this capacity is lost in RBC units after 2 or 3 weeks of storage [20] and this loss is not reversible after 3 weeks of storage [21]. Thus, possible mechanisms of transfusion-related thrombosis include lower potential of deformability, higher adherence and aggregation of RBCs, associated vasoconstriction, or the presence of a procoagulant substance (related to lipid release) in the stored supernatant of RBCs stored for longer than 21 days. However, increased adherence of RBCs to endothelial cells is demonstrated in stored RBC units and may be mediated by the RBC membrane change that increases with time. RBC surface phosphatidyl-serine has also been reported to cause platelet activation and aggregation, which can be prothrombotic [22,23]. However, the increased adherence of stored RBC can be abolished by pre-storage leuko-reduction [24].

The interaction between intra-erythrocyte hemoglobin and nitric oxide (NO) is also disturbed in older RBC units [25]. Normal RBCs react almost immediately to local hypoxia by releasing NO: this causes regional vasodilatation. Conversely, RBCs retain NO if local oxygen content is adequate: this causes regional vasoconstriction. This function is disturbed within 3 hours of storage [20]. Moreover, free hemoglobin is released in the supernatant of stored RBC units [20]. This free hemoglobin has the potential to retain NO. Thus, older RBC units may retain NO, causing vasoconstriction and venous stasis, which may increase the risk of thrombosis.

Indeed, strong laboratory data suggest that storage lesion may increase VTE risk after the transfusion of older RBC units. Conversely, clinical evidence supporting an association between RBC age or RBC transfusion (or both) and DVT is weaker. RBC rheology (deformability and aggregation) is disturbed in patients with sepsis, and deteriorating rheology is associated with increased mortality in patients with sepsis [26].

Strengths of our study include consecutive enrollment of medical or surgical ICU patients, making selection bias unlikely. We enrolled heterogeneous medical and surgical critically ill patients, as opposed to solely trauma patients (which have been previously studied [12,14]) and this increases the generalizability of our findings. Transfusion practices were stable over the year of the study. We considered a broader range of RBC transfusion volume in comparison with one earlier study [14] in which only patients transfused more than 5 units were analyzed. There was a systematic and prospective monitoring of DVT incidence and this should prevent any detection bias. DVTs were diagnosed by ultrasound, which is more reliable than diagnosis based on clinical symptoms [27]. Each screening ultrasound was conducted independently by credentialed sonographers who were blinded to patient characteristics and who were previously shown to have excellent intra-rater reliability [16]. We adjusted for four previously identified independent predictors of DVT [17] in a rigorous multivariable analysis.

## Conclusions

Although higher numbers of transfused RBC units and prolonged storage of RBCs have been associated with increased risk of DVT in trauma patients, we did not observe this association in medical or surgical ICU patients. Our results do not suggest the need to decrease the number of transfused units or shorten RBC storage duration when transfusing RBCs in medical or surgical critically ill patients. Further studies are needed to more thoroughly evaluate the possible relationship between RBC transfusions and VTE in critically ill patients. Such studies include ABLE (The Age of Blood Evaluation Trial) (ISRCTN44878718), an ongoing international randomized trial comparing younger RBCs (stored fewer than 7 days) with standard-issue RBCs.

## Key messages

• Inflammatory membrane lipid changes on red blood cells (RBCs) stored for prolonged periods facilitate thrombin generation, which may lead to a potential hypercoagulable state.

• Although higher numbers of transfused RBC units and prolonged storage of RBCs have been associated with increased risk of deep vein thrombosis (DVT) in trauma patients, we did not observe this association in medical or surgical intensive care unit patients.

• In our study, RBCs stored for fewer than 7 days were associated with a trend toward a higher risk of DVT compared with RBCs stored more than 7 days.

## Abbreviations

APACHE II: Acute Physiology and Chronic Health Evaluation II; CI: confidence interval; DVT: deep vein thrombosis; ICU: intensive care unit; NO: nitrous oxide; RBC: red blood cell; VTE: venous thromboembolism.

## Competing interests

The authors declare that they have no competing interests.

## Authors' contributions

CK carried out the data acquisition and secondary database construction, obtained the funding, and drafted the manuscript. LG participated in the design of the study, helped to draft the manuscript, and performed the statistical analysis. DC conceived of the study and participated in its design and coordination and helped to draft the manuscript. PS, JL, MC, PH, MM, WG, and CR helped to draft the manuscript. All authors read and approved the final manuscript.

## References

[B1] Corwin HL Gettinger A Pearl RG Fink MP Levy MM Abraham E MacIntyre NR Shabot MM Duh MS Shapiro MJ The CRIT Study: anemia and blood transfusion in the critically ill--current clinical practice in the United States Crit Care Med 2004 32 39 52 10.1097/01.CCM.0000104112.34142.79 14707558

[B2] Raghavan M Marik PE Anemia, allogenic blood transfusion, and immunomodulation in the critically ill Chest 2005 127 295 307 10.1378/chest.127.1.295 15653997

[B3] Hebert PC Wells G Blajchman MA Marshall J Martin C Pagliarello G Tweeddale M Schweitzer I Yetisir E A multicenter, randomized, controlled clinical trial of transfusion requirements in critical care N Engl J Med 1999 340 409 417 10.1056/NEJM199902113400601 9971864

[B4] Marik PE Corwin HL Efficacy of red blood cell transfusion in the critically ill: a systematic review of the literature Crit Care Med 2008 36 2667 2674 10.1097/CCM.0b013e3181844677 18679112

[B5] Oliver E Carrio ML Rodríguez-Castro D Javierre C Farrero E Torrado H Castells E Ventura JL Relationships among haemoglobin level, packed red cell transfusion and clinical outcomes in patients after cardiac surgery Int Care Med 2009 35 1548 1555 10.1007/s00134-009-1526-0 19547956

[B6] Vamvakas EC Pneumonia as a complication of blood product transfusion in the critically ill: transfusion-related immunomodulation (TRIM) Crit Care Med 2006 34 151 159 10.1097/01.CCM.0000190619.42013.94 16617260

[B7] Tinmouth A Fergusson D Chin Yee I Hébert PC ABLE Investigators Canadian Critical Care Trials Group Clinical consequences of red cell storage in the critically ill Transfusion 2006 46 2014 2027 10.1111/j.1537-2995.2006.01026.x 17076859

[B8] Yoshida T AuBuchon JP Tryzelaar L Foster KY Bitensky MW Extended storage of red blood cells under anaerobic conditions Vox Sang 2007 92 22 31 10.1111/j.1423-0410.2006.00860.x 17181587

[B9] Cardo LJ Hmel P Wilder D Stored packed red blood cells contain a procoagulant phospholipid reducible by leukodepletion filters and washing Transfus Apher Sci 2008 38 141 147 10.1016/j.transci.2007.09.006 18346937

[B10] Tissot JD Rubin O Canellini G Analysis and clinical relevance of microparticles from red blood cells Curr Opin Heme 2010 17 571 577 10.1097/MOH.0b013e32833ec217 20960973

[B11] Napolitano LM Corwin HL Efficacy of red blood cell transfusion in the critically ill Crit Care Clin 2004 20 255 268 10.1016/j.ccc.2003.12.002 15135464

[B12] Geerts WH Code K Jay R Chen E Szalai JP A prospective study of venous thromboembolism after major trauma N Engl J Med 1994 331 1601 1606 10.1056/NEJM199412153312401 7969340

[B13] Weingberg JA McGwin G Vandromme MJ Marques MB Melton SM Reiff DA Kerby JD Rue LW Duration of red cell storage influences mortality after trauma Trauma 2010 69 1427 1432 10.1097/TA.0b013e3181fa0019 PMC3136808 21150522

[B14] Spinella P Carroll C Staff I Gross R Mc Quay J Keibel L Wade CE Holcomb JB Duration of red blood cell storage is associated with increased incidence of deep vein thrombosis and in-hospital mortality in patients with traumatic injuries Crit Care 2009 13 R151 162 10.1186/cc8050 19772604 PMC2784373

[B15] Fontaine MJ Chung YT Erhun F Goodnough LT Age of blood as a limitation for transfusion: potential impact on blood inventory and availability Transfusion 2010 50 2233 2239 10.1111/j.1537-2995.2010.02690.x 20497519

[B16] Cook DJ Medic A Andrews J Doppler compression ultrasound of the lower extremities in ICU patients: an inter-rater reliability study Crit Care Med 2003 Suppl A 346

[B17] Cook DJ Crowther M Meade M Rabbat C Griffith L Schiff D Geerts W Guyatt G Deep venous thrombosis in medical-surgical critically ill patients: prevalence, incidence and risk factors Crit Care Med 2005 33 1565 1571 10.1097/01.CCM.0000171207.95319.B2 16003063

[B18] Cook DJ McMullin J Hodder R Heule M Pinilla J Dodek P Stewart T Canadian ICU Directors Group Prevention and diagnosis of venous thromboembolism in critically ill patients: a Canadian survey Crit Care 2001 5 336 342 10.1186/cc1066 11737922 PMC83855

[B19] Hirsch DR Ingenito EP Goldhaber SZ Prevalence of deep venous thrombosis among patients in a medical intensive care unit JAMA 1995 274 335 337 10.1001/jama.274.4.335 7609264

[B20] Bennett-Guerrero E Veldman TH Doctor A Telen MJ Ortel TL Reid TS Mulherin MA Zhu H Buck RD Califf RM McMahon TJ Evolution of adverse changes in stored RBCs Proc Natl Acad Sci USA 2007 104 17063 17068 10.1073/pnas.0708160104 17940021 PMC2040393

[B21] Bosman GJ Werre JM Willekens FL Novotný VM Erythrocyte ageing in vivo and in vitro: structural aspects and implications for transfusion Transf Med 2008 18 335 347 10.1111/j.1365-3148.2008.00892.x 19140816

[B22] Koshkaryev A Zelig O Manny N Yedgar S Barshtein G Rejuvenation treatment of stored red blood cells reverses storage-induced adhesion to vascular endothelial cells Transfusion 2009 49 2136 2143 10.1111/j.1537-2995.2009.02251.x 19538542

[B23] Sweeney J Kouttab N Kurtis J Stored red blood cell supernatant facilitates thrombin generation Transfusion 2009 49 1569 1579 10.1111/j.1537-2995.2009.02196.x 19413726

[B24] Chin-Yee IH Gray-Statchuk L Milkovich S Ellis CG Transfusion of stored red blood cells adhere in the rat microvasculature Transfusion 2009 49 2304 2310 10.1111/j.1537-2995.2009.02315.x 19624601

[B25] Doctor A Platt R Sheram ML Eischeid A McMahon T Maxey T Doherty J Axelrod M Kline J Gurka M Gow A Gaston B Hemoglobin conformation couples erythrocyte S-nitrosothiol content to O_2 _gradients Proc Natl Acad Sci USA 2005 102 5709 5714 10.1073/pnas.0407490102 15824313 PMC556285

[B26] Donadello K Reggiori G Vincent JL Piagnerelli M La détérioration progressive de la déformabilité érythrocytaire au cours du sepsis est associée à une mortalité accrue. (Progressive deterioration of erythrocyte deformability in sepsis is associated with increased mortality.) Réanimation 2010 19 S93

[B27] Kieron CJ Julian JA Newman TE Ginsburg JS Noninvasive diagnosis of deep venous thrombosis. McMaster Diagnostic Imaging Practice Guidelines Initiative Ann Intern Med 1998 128 663 677 9537941 10.7326/0003-4819-128-8-199804150-00011

